# Self-regulation in Barth syndrome: a qualitative perspective of adolescents, adults and parents in the U.K

**DOI:** 10.1186/s13023-021-02027-5

**Published:** 2021-09-29

**Authors:** Aidan Searle, Georgia Herbert, Lucy Dabner, Colin G. Steward, Michaela Damin, Guido Pieles

**Affiliations:** 1 National Institute of Health Research (NIHR) Bristol Biomedical Research Centre (Nutrition Theme), Education and Research Centre, University of Bristol, Upper Maudlin Street, Bristol, BS2 8AE UK; 2 Clinical Trials and Evaluation Unit, Bristol Trials Centre, Bristol Medical School, University of Bristol, Bristol, UK; 3 School of Cellular and Molecular Medicine, Biomedical Sciences Building, University of Bristol, University Walk, Bristol, BS8 1TD UK; 4 Barth Syndrome UK, 1 The Vikings, Romsey, SO51 5RG Hampshire UK; 5 Bristol Congenital Heart Centre, University Hospitals Bristol NHS Foundation, Bristol, UK; 6 National Institute of Health Research (NIHR) Biomedical Research Centre (Cardiovascular Theme), University Hospitals Bristol NHS Foundation Trust and University of Bristol, Bristol, UK

**Keywords:** Barth syndrome, Male, Self-regulation, Qualitative

## Abstract

**Background:**

Barth syndrome (BS) is a life-threatening genetic disease caused by abnormal lipids in the mitochondria of cells and mostly affects young males. Those living with BS have severe exercise intolerance, lethargy and fatigue due to muscle disease which affect their daily life. Previous research suggests a need for qualitative exploration of self-regulation in BS and the inter-personal processes at play in family life. Therefore this study aimed to explore self-regulation and coping strategies and inter-personal responses in individuals and families affected by Barth syndrome. A multi-perspective qualitative study based on face to face, semi-structured, in-depth interviews with 11 participants (9–27 years, mean 15 years) with BS and/or their parents participating in a randomised double-blind clinical drug trial (CARDIOMAN). Interviews were transcribed verbatim and managed in NVivo prior to conducting a thematic analysis (AS and GH).

**Results:**

Four key themes were identified: diagnosis and treatment, social support, identity and social integration, symptoms and self-regulation. The present findings suggest that self-regulation and coping in boys with BS was interpersonal and contingent on parental awareness such that parents were aware that their child had a limited energy reserve and that had to be managed due to the implications of fatigue for daily living.

**Conclusion:**

The findings support previous quantitative work demonstrating that children and parents tend to share a coherent view of BS. However, there is a need for greater awareness from others within the wider context of social and employment networks to minimise adverse implications for future life choices.

## Introduction

Barth syndrome is a life-threatening genetic disease caused by abnormal lipids in the mitochondria of cells, mostly affecting young males. At present, there are twenty-seven patients with the disease treated in the U.K. Barth Syndrome Service. The presentation of Barth syndrome (MIM 302060) typically includes skeletal muscle weakness, neutropenia, and growth retardation [1, 2]. In addition, two metabolic abnormalities are typically present; elevated urinary excretion of 3-methylglutaconic acid and hypocholesterolemia [2]. Furthermore, a mild cognitive phenotype has been described [3] and there is individual variability in the age of onset, the expression of symptoms, and progression of the disease.

Those affected can develop heart failure during the first decade of life and can struggle with poor growth or feeding problems during childhood. Serious bacterial infection is another life-threatening issue due to low blood neutrophil counts in 90% of patients [4]. As a result, two thirds of UK patients require chronic subcutaneous injection therapy with granulocyte colony stimulating factor (G-CSF), a distressing and expensive medication provided by the UK National Health Service (NHS). They often remain intermittently neutropenic despite receiving G-CSF.

There can be rapid deterioration during periods of stable health and even when under expert medical care. Ventricular arrhythmia (tachycardia or fibrillation) affects 10% of adolescents and can cause sudden cardiac death at any stage of childhood, including the neonatal period [5]. These seemingly random acute crises are not predictable by genotype or recent medical history, producing a need for cardiac resuscitation training, use of automated external defibrillators and/or implanted cardiac reveal devices, and causing chronic anxiety in affected families. Twenty-two percent of boys living in the UK have undergone cardiac transplantation and others require ongoing management for cardiomyopathy, yet patients continue to die from this disease despite best conventional therapy.

### Psychosocial impact in Barth Syndrome

Those living with BS experience severe exercise intolerance, lethargy and fatigue which affects their daily life. Lethargy and fatigue can interfere with schoolwork and play and often necessitates the use of wheelchairs. Similarly, patients are compromised in their ability to hold down strenuous or demanding jobs which impacts quality of life and ability to obtain employment [6].

Patients and their families often experience many daily challenges associated with the disease, as well as major healthcare costs associated with transplantation, medications and limitations around employment.

The management of neutropenia in this disease is challenging since patients have highly variable neutrophil counts, preventing administration of a consistent daily dose and requiring repeated blood counts and clinical management. Recurrent subcutaneous injections and repetitive blood counts can produce needle phobia and compliance issues.

Previous quantitative approaches to measuring quality of life and the psychosocial impact of living with BS have been conducted in the US. For example, Storch et al. [7] found that self-reported quality of life was lower in young people with BS compared with healthy peers; they were also more socially isolated and displayed less independent functioning than healthy peers. In addition, their parents saw a need for academic assistance for their child. A pertinent aspect of this study is that young people with Barth Syndrome rated their own psychosocial functioning in a way that was consistent with their parents’ rating. Such parent–child coherence is not typically seen in families of children with chronic illness, where parents typically report more observed problems than those reported by their child [8].

A further study showed that individuals with BS exhibited poorer quality of life, physical functioning and social functioning than individuals with diabetes and cancer, based on both parental and child reports [9]. This study also measured the psychological functioning in parents of individuals with BS with parents reporting higher ‘internalised’ care-giver strain, such as negative feelings, compared to objective or ‘externalised’ strain such as disruptions to daily life like schedule changes. Furthermore, parents were adaptive rather than maladaptive in their response to their child’s illness and demonstrated acceptance, positive reframing, emotional support and instrumental support. These observations are a realistic reflection of caregiver anxieties regarding a child’s prognosis and long-term health concerns.

### Self-regulation and coping

Self-regulation is defined as the ability to modulate cognition, emotion and behaviour toward a goal and includes both individual and interpersonal goals [10]. Self-regulation in chronic illness includes interpersonal processes such as the ability to use interpersonal resources in their environment [11]. Past research suggests that adolescents must also regulate their cognitive, emotional, and behavioural responses to unrequested parental involvement in disease management, such as parental monitoring, disclosure and family conflict [12]. Typically, the interpersonal context in paediatric research is characterised in the way that parents, peers, and health professionals influence disease management. Evidence for the importance of adolescents’ self-regulation skills in these interpersonal processes comes from studies showing that parental involvement and knowledge are actually reflective of how adolescents involve their parents in chronic illness management [13].

### Self-regulation and coping strategies in Barth syndrome

Coping strategies are also defined as the cognitive, emotional and behavioural means by which individuals manage the symptoms and daily living with a condition [14]. To date there is little research that has qualitatively explored the lived experience and coping strategies in BS. However, (Mazar et al. [15] explored the experience of living with BS from the perspective of older adult males (above 35 years of age). This study mostly focused on signs and symptoms of BS but also evaluated coping strategies. Individuals’ coping strategies included adapting daily routines, managing physical limitations and emotional responses. Signs and symptoms were found to impact negatively on individuals’ emotional, physical, social and role functioning from an early age into adulthood.

The present qualitative research is based within the context of a clinical trial in which the participant and family experience of BS and their participation in the trial were also documented. This embedded qualitative research enabled the authors to explore self-regulation and coping and the inter-personal responses in individuals and families affected by Barth syndrome.

## Methods

### Ethical approval

Ethical approval for the qualitative interview study was granted as part of the clinical trial within which it was conducted (REC ref: 15/SW/0228) on 12/11/2015.

### Participants

The participants were in a double blind, crossover, randomised controlled trial of the oral drug bezafibrate (CARDIOMAN trial; see “Appendix 1” for selection criteria) and consisted of 7 males aged 14 years and above and 4 males below 14 years. Participants aged 14 and above were interviewed, along with the attending parents of all participants. The interview data formed the basis of evaluating trial participation as well as gaining insight into living with the disease. All patients are under the care of the national NHS Specialised Services Barth Syndrome Service, which is based at Bristol Royal Hospital for Children. Participants and their families attend this service from across the UK.

### Topic guides

Topic guides were developed for interviews conducted at both phases of the crossover trial (4 and 9 months) based on discussions with the study team and grant co-applicants, which include medical specialists.

The topic guide for phase 1 included items in relation to: family, schooling, occupation, diagnosis of BS, symptoms of BS, previous and current treatment for BS, living with BS and physical impact, quality of life, social and psychological impact of BS, support, adherence and/or issues with medication and engagement with medical practitioners.

The topic guide for phase 2 of the trial was limited to: symptoms of BS, experience of the trial, quality of life, social and psychological impact of BS, adherence and/or issues with medication and engagement with medical practitioners.

### Interviews

Face-to-face semi-structured interviews were conducted with seven participants in the CARDIOMAN trial aged 14 and above, and four interviews with parents of those aged under 14 years (see Table 1 for details of interview context). All interviews were conducted in a private room whilst families were attending a clinical assessment unit for the study. Written informed consent for all interviews was obtained prior to the first phase of interviews. Parents of children gave their consent for their own and their child’s participation but children under 14 were not interviewed (although were in the room as their parent/s were interviewed). Participants aged 14 years and above were interviewed alone or with a parent if preferred, and two adult participants were interviewed alone. A total of 22 interviews were conducted at two time-points: following the first treatment phase (at 4 months) and at the end of phase 2 (at 9 months).

**Table 1:** Details of participants and interview schedule

ID	Phase 1—month (interviewed with)	Phase 2—month (interviewed with)	Total interviews
01	Alone	Alone	2
02*	Mother/father	Father	2
03	Alone	Alone	2
04	Mother	Mother	2
05	Alone	Alone	2
06	Alone	Father	2
07*	Mother/father	Mother	2
08*	Mother/father	Father	2
09	Father	Father	2
010*	Mother	Mother	2
011	Mother	Mother	2
No. interviewees	4 interviewed alone, 7 with parent/s	3 interviewed alone, 8 with parent/s	22

Age range 9–27, mean 15 years

^*^Under 14 years of age at study entry, interviews conducted with parents

### Analysis

All the interviews were audio-recorded and transcribed verbatim. A sample of six transcripts representing both time-points were read and re-read by two experienced qualitative researchers (AS and GH) to familiarise themselves with the data. AS and GH then met to discuss their overall impressions of the whole dataset and an inductive thematic analysis was conducted on both data sets [16]. Both AS and GH independently coded the transcripts and then met again to discuss their coding and to develop coding frames for the complete dataset. Having done so, AS and GH coded transcripts using an agreed coding frame and met again to verify coding. At each phase there were codes that were specific to participants’/families’ experiences of living with BS. These codes included: Living with BS, Symptoms of BS, Treatment of BS, Child Development and Growth, Communication and Role of MDT, Social Functioning and Integration, View of Self, Schooling and BS, Social Support, Psychological Support for BS, Perceived Stigma and Normalisation, and Management and Coping Strategies. Once each coding frame was finalised, all the transcripts were imported into NVivo 12 and electronically coded.

### Key themes

**Fig. 1:**
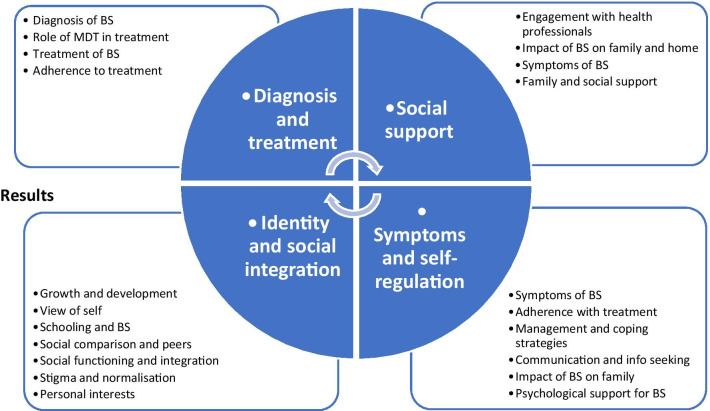
Key themes and codes representing self-regulation and coping in Barth Syndrome

The analysts met again and endeavoured to reach consensus about how the individual codes informed the development of themes from within the dataset (Fig. 1). This process led to identifying 4 key themes:

- Diagnosis and treatment
- Social support
- Identity and social integration
- Symptoms and self-regulation

## Results

### Diagnosis and treatment

Parents reflected on their sons’ diagnosis of BS and knowledge at this time was important for parents to understand the condition as a syndrome—i.e. that the heart was not the only organ affected by the condition. They understood that BS could be life-threatening but were reassured by knowing their sons were getting the correct care:

*So, to me, his immune system, that’s just as dangerous or as important as the heart. So, having that diagnosed as well and knowing that all these things that were going on with [Name] were then umbrellaed under the syndrome Barth, it gave absolute clarity, and obviously completely then added a safeguard to [Name], since we were getting the correct care. We were getting all this knowledge and all this information coming at us, which, you just kind of go, outside of the diagnosis.* (011, Phase 1, Mother)

*I think it's great when you're first diagnosed and you're really in that shock phase when it's quite scary and you don't know what's going on. Why is your kid behaving like this and why is your kid manifesting this, that and the other? So you're really, really wanting to have all that information. A lot of the stuff that comes up perennially on this, like feeding issues...* (02, Phase 1, Father)

Parents became aware of individual differences in the symptomatology of BS and of the potential risks the condition might pose for their child in the future, such as the risk of heart failure. In addition, they were made aware of the extent that medication was managing the condition in the present and would be necessary throughout their lives:

*`There are different levels, there are different abilities, some children have had organ transplants, you know. [Name] hasn’t had that, obviously he’s got cardiomyopathy, but as much as he’s got cardiomyopathy, his heart, at the moment, is functioning as a normal poor-functioning heart. I think he’s doing really well, you know, happy, healthy and on a lot of meds. I do worry about how those meds will impact him in the future, but there’s no point me worrying about that at the moment, because he’s got to take the meds. So, it’s just a balance.* (011, Phase 1, Mother)

*Even before they diagnosed the Barth syndrome, they said, "These drugs, he'll be on this for life." I was saying, "Do we have to stop them doing sports?" They said, "Oh, no, they can carry on doing sports as far as they're able," and this, that and the other, "They'll never be professional sportsmen, but they'll be on these drugs for the rest of their lives."* (06, Phase 1, Father)

### Social support

Individuals and families spoke of the emotional and instrumental support they received from knowing each other through the community, within the clinic and involvement in national and international support networks. An example of emotional support:

*Our community is very, very (laughter) close. Like, we all know each other. There are certain people we know. You could name any… because it’s just a very rare disease. There are like 200 of us known, only. And …there are certain people you could just go- and it’s like, you could just say- you could pull out any name randomly and if it’s an affected individual, she’s like, ‘Oh, yes. That person lives over there in America,’ wherever they are, and it’s just… we’re very close, and very tight. We’re like a family, and it’s really nice.* (03, Phase 1)

The networks provided opportunities to share experiences with other families and highlighted that parents and children demonstrated instrumental support with each other by managing the condition through sharing information:

*It's useful for some of the feedback because, when they do meet up once a year and have these gatherings, a lot of parents then talk to each other and actually share common problems with their children, where they're able to discuss and say, ‘Oh, we were told to try this’ and it's learning from learning, second-degree learning, that is passed on from the team here. So those things are always useful.* (08, Phase 1, Father)

However, despite facing difficulties themselves one family found consulting support groups for BS to be a negative experience. For example, it was seen as relative, such that if their child was doing well it was not helpful to learn of others’ misfortune such as bereavement:

*So, to me, after everything we’ve gone through, with his heart having been so weak and leaks and all… being, really, so overgrown and stretched for his body, where he’s at even with that, under the cardiomyopathy angle, he’s still in a really good place. So, I would rather go half-full, positive, than keep on thinking about the negatives and what… and I find that looking at those groups can often walk me back and you kind of go, ‘Somebody’s had a bereavement,’ and it’s horrendous. But my ignorance keeps me strong, if I’m honest.* (011, Phase 1, Mother)

There was also a temporal element so that some families felt support groups were more helpful in the early stages of diagnosis rather than the adolescent stage:

*I think it would have been nice to have an outlet, yes, away from the family outlet, because you're always trying to be strong for each other and everything else and put up this image, but that doesn't necessarily relate to how your feelings are. Yes, I guess it would have been useful at the beginning, but that was what, 12, 14 years ago now so.* (08, Phase 1, Father)

However, having early support was seen to have benefitted adolescent boys in the longer term to with regard to asking for help:

*I would say [Name] is very good at speaking up for himself and telling people what he needs, so psychologically, it's been good in the sense that you can speak up for yourself. Would you say? Are you speaking up for yourself or speaking for...? .* (02, Phase 1, Father)

*Now they’ve changed the clinics here down to micro-clinics, the ability for families to get together has been very much lost. We haven’t done a family gathering now for two years, which is unusual, it used to be yearly. So, we do keep in touch on Facebook and that sort of thing, but the ability for the boys to get together and the parents to get together in a group and all of us to sit down and have a chat, doesn’t happen very often anymore. So, most of our support comes out of either Children’s Hospice or out of the [Name] nurses*.” (010, Phase 1, Mother)

### Identity and social integration

Parents were concerned that their sons should not be singled out on the basis of having BS. However, this could be paradoxical in that attempts at integration in school sports events and /or social activities could have a negative experience for boys if poorly managed:

*Well, I don’t want him identified by the syndrome. Yes, I want it recognised when it needs to be recognised, but I think [Name] manages very well and it’s only if he’s not managing do we kind of go, ‘Right, can we be clear, these are the elements that need to be considered.’ Like the sports day, I’ll be damned if I ever let the school put him in that position again and that was even talking to them at length prior to this sports day. It was utterly horrible, emotionally for [Name] and horrible to watch.* (011, Phase 1, Mother)

Furthermore, as sporting ability is strongly associated with social acceptance and integration in adolescence, a perceived lack by others of ability to compete was felt to be socially and psychologically damaging for some boys and resulted in a lack of inclusion:

*He used to come home and say, about playing football—which is obviously why he hasn't got a big circle of friends—‘I can't play football, they won't kick the ball to me.’ ‘Well, no, you've got to train.’ He can't compete in that sort of… So, you don't know if it is because of Barth syndrome, so you don't know if that's what's made him like he is today, because he's very quiet, isn't he, [Name]? Whereas [brother]'s a bit more outgoing.”* (06, Phase 1, Father)

*Sports day was horrendous, and always has been horrendous, at the end of year. And, because he’s not in a wheelchair, they overlook the disability, and they left him just sitting on the grass for too long on his own, just becoming withdrawn until he sat there crying his eyes out while everybody else is just running around. So, I went across and I just pulled him out and just went, ‘We’ll go to the coffee shop’.* (011, Phase 1, Mother)

Nonetheless, parents of boys with BS were aware of the importance of participating in school sports activities. Parents encouraged their child to participate despite their disadvantaged position in an attempt to normalise, rather than stigmatise, their child:

*They did a cross country, the school, and it was just under two miles. I think it was about two miles. The first kid came in at just under seven minutes and beat the school record….. So, the first time they didn't make [Name] do it and I rang his school because [Name] and I were like, ‘Actually, that's wrong. He should have been made to do it. Even if he failed, the point is he should have taken part.’ ….we rang them, so the next time they did it they invited [Name] to join in and he did it. So, he walked it. It took him half an hour but he said it was like the best day of his life. He walked into school; teachers came out of the classroom to high five*. (07, Phase 1, Mother)

However, non-participation in sport could mean watching on the side-lines which could be disheartening for parents as well as the child:

*To watch football, yes. He would love to play, and I think that does have some psychological, emotional things for him. It also, I guess, has it for us as well in terms of looking and seeing that he wants to do something and he's physically not able to. So that's disheartening to an extent.* (08, Phase 1, Father)

The weakness and fatigue associated with BS due to compromised heart function could mark out boys in the school environment as they were more vulnerable to risk:

*So, if he gets knocked and it’s… they’re just kids brushing past quickly he’s much likelier to tumble than anybody else. We make sure that he doesn’t have to carry his school bag round because it would add extra fatigue with the weight, he could tumble again more. So, I’ll always hand his school bag into his classroom. Little things…*(011, Mother, Phase 1).

Another issue associated with fatigue was that it is poorly understood by others in their social environment and they are thus labelled as ‘lazy’:

*Because there are heart condition aspects, but my heart is stable and I’m doing okay, thankfully. But, yes, fatigue- I’m tired all the time. It’s difficult because everybody labels you as lazy and you don’t want to do anything. I’m like, “Yes, because I’m tired. That’s why I don’t want to do anything.”* (03, Phase 1)

Future oriented social integration was a cause for concern for parents and some had started to consider how their son’s futures would look. As established friendships become more fluid, due to life changes and educational choices, the ability to initiate new friendships could present a challenge:

*Oh, yes, they have got to start thinking about their futures. He was just so little and we just let him carry on with it. With [Name], he was happy and healthy in and of himself, but I think he'd be happier if he could get out a bit more, have the ability to do what other friends do, or other people he's known. They've both got a very small circle of friends. Both of them find it hard to make new friends. [Name] is beginning to come out of himself a little bit more now, ‘I can go to uni.* (06, Phase 1, Father)

There were also clinical and biological factors that some parents perceived would increase their child’s risk of infection and imposed restrictions on large social events for their son with BS. In particular this was due to neutropenia and compromised immune functioning:

*He’s neutropenic, so obviously that has a slight pressure on you because you do have to rethink things sometimes because you're not going to go into a place where it's thousands of people and go to a festival. You know that he could potentially pick something up, so you're a little bit more careful, but we try and do most things.* (08, Phase 1, Mother)

### Symptoms and self-regulation

Fatigue was a prompt for self-regulation. However, some boys expressed that their fatigue could be overwhelming:

*Well, I wouldn’t say it’s done my mental health any good. Well, as unhealthy as it is, I just sort of try to avoid thinking about the problems and just, you know, try and carry on as best you can. It’s like, ‘Yes, I’m tired. Okay. I will do less today,’ or, like, ‘Oh, I’m tired, again.’ It’s like, ‘Yes.’ It’s like, at a point where if you get down about it- Yes, you have days when you feel down because… Well, it sucks. Big time.* (03, Phase 1)

Some boys self-regulated by employing behavioural coping strategies such as restricting their time with friends or asking a teacher to sanction time out of class:

*I always try to make a good social effort. At college, sometimes I'm having to leave mates so I can go and sleep so I can manage the next day. But I think that's normal and everybody has to do that from time to time, so it doesn't really bother me that much.”* (03, Phase 2)

Parents were also aware that their child could challenge themselves with regard to onset of fatigue knowing they could be autonomous in their attempts at self-regulation:

*I think we try to be as normal with everything as we can, which in turn gets him to do more. So he challenges himself on quite a few things and even though he's tired he'll say, "Oh, can we rest for a bit?", and then five minutes later, "Right, let's go." So he does push himself. He doesn't wallow in self-pity, which is great, and he's just a delightful, focused little kid. All the challenges that you could have attributed to a condition like this, I think that he just goes past most of them on his own accord without being pushed or anything.* (08, Phase 1, Father)

Parents also thought that participation in social events could be managed but this had implications in terms of physical recovery:

*Yes, the energy cells so that you have, "Oh yes, you're great. You're doing fine," and then 'bang'. If you were playing rounders, you might do a one to one, so physically... If you're a kid at a sleepover, a couple of days later, you might be tired in that way much more. His coordination and recovery period are, yes, very different. The sleep was good. You can see the core-stability thing. You can know by a kid.* (02, Phase 1, Mother)

Some parents felt compelled to intervene in situations where the child was engaged in physical activity such as physical activity prescription schemes. The parents also encouraged their child to advocate for themselves:

*As well as tailoring the exercises to what [Name] can do, rather than what he'd like him to do. So, he's taken into account… We went in to the interview with him, told him what was likely to happen if he was having trouble, any difficulties. [Name] is of an age now where he should, himself, be able to say, "Look, I've got to stop.* (06, Phase 1, Mother)

*We told him, when you feel… Instead of saying, “Go on, go on” you've got to turn around and say “I can't, I need to rest.” Whereas, before, he would carry on, now he's more mature. (*06, Phase 1, Father)

There also appeared to be a degree of day to day variation in energy but despite parental experience it could not be monitored within a known range:

*It’s very up and down, I think, because there are times when he can participate a lot more than others. It’s weird because I think, with the condition, you never know what you're going to get, day by day. It’s not something that you know, “Okay, this is the benchmark, he’s going to be able to walk 500 steps a day maximum, minimum 300” so you’ve kind of got a range to go with*. (08, Phase 2, Father)

Some behavioural coping strategies were endorsed by parents in the school environment to negate negative peer to peer and/or social comparison:

*He doesn’t perceive himself as different from anybody else at all. He goes to a small school where they do horizontal age grouping, so the children within his school setting are from three and a half up to twelve. So, it’s mixed age groups, so therefore he doesn’t really perceive himself as any different to anybody else physically.* (010, Phase 1, Mother)

## Discussion

This is the first qualitative exploration of the experience of individuals born and living with Barth Syndrome (BS) from the perspective of adolescents, young adults and parents. The findings suggest that self-regulation and coping in boys with BS was interpersonal and contingent on parental awareness, such that parents were aware that their child had a limited energy reserve and that had to be managed due to the implications of fatigue for daily living. Furthermore, individual differences in the impact that these participants reported was rooted in the same set of symptoms (tiredness, weakness) although the level of impact was dependent on the level of perceived symptom severity.

### Findings in context of previous research

The observations represent parental anxieties regarding their child’s prognosis and long-term health, educational and vocational concerns. These findings also resonate with Jacob et al. [9] where parents of individuals with BS reported higher ‘internalised’ care-giver strain (e.g. negative feelings) compared to objective or ‘externalised’ strain (e.g. disruptions to daily life, such as schedule changes). However, an encouraging finding of the present study is that parents used adaptive rather than maladaptive coping strategies in response to their child’s condition, such as acceptance, positive reframing, emotional support and instrumental support, which were manifested through inter-personal relations, support groups, and medical professionals. Furthermore, there was an observed inter-dependency, demonstrating that the management of BS is dependent on both adolescent and parents. Previous research has shown that the efficacy of adolescents’ self-regulation skills in these interpersonal processes is driven by parental involvement and their level of knowledge, and is reflective of how adolescents involve their parents in disease management [13]. Indeed, research in the health behaviours of adolescents suggests that many self-regulation skills can be recast as both individual and interpersonal in nature. For example, adolescents must regulate their cognitive, emotional and behavioural responses to unrequested parental involvement in disease management and foster a sense of autonomy, which may lead to conflict [12]. However, there was little evidence of family conflict in the present data set suggesting that coping strategies were carefully implemented and demonstrated parent–child coherence that resonates with the findings of Williams et al. [8]. The present data also appear to support those of Storch et al. [4] in that self-regulation and psychosocial functioning in young people with Barth Syndrome is coherent within families; parents showed a high level of awareness of their son’s limitations and the impact of BS on their school and social lives.

Qualitative research [15] found that, for adults living with BS, coping strategies included adapting daily routines, managing physical limitations and relying on social support networks. The present study found that the development of these skills in adolescents with BS is mediated by external forces; many boys were still developing emotionally and physically and within the structure provided by full-time education and family living. In the Mazar et al. study [15], participants reported that their symptom progression increasingly limited their physical functioning and independent living thus there is a need for greater awareness from others within the context of social and employment networks in the absence of family support. Finally, there are implications for care and future research arising from these qualitative findings such that it should be considered how coping strategies can be implemented for long-term role function, employability and independent living. These findings, from individuals below the age of 35, help to give psychosocial context to the natural history and progression of BS, and demonstrate the resilience of patients and their adaptation to BS.

### Data quality and reflexivity

A strength of this study is that data was collected via interviews conducted at 2 time-points. This longitudinal approach optimised rapport and candour with participants and their parents over 5 months. Furthermore, the multi-perspective approach serves to better understand how adolescents, young adults and their parents experience BS within the context of family life. The density of the data and the consolidation of emerging themes suggest that data saturation was achieved.

The lead author and analyst (AS) has extensive experience of qualitative research in patients’ experience of illness and engagement with health services but had no prior experience of BS or working within the NHS Specialised Services Barth Syndrome Service in the U.K. Similarly, the second data analyst (GH) is also an experienced qualitative researcher with no prior experience of BS or its treatment, which serves to enhance the robustness of the findings and minimises the risk of inherent bias.

From a historical and contextual perspective, it was important to patients attending the BS service that there was access to a playroom to act as a social hub where patients and parents could socialise whilst waiting for investigations and medical reviews. The MDT at the service also led teaching sessions about potential outcomes such as heart failure or neutropenia. This opportunity for socialising and learning served in providing cohesion between patients and families. In addition, previous clinics run by the Barth Syndrome Service were of longer duration, which facilitated greater face-to-face time with both medical professionals and with other families. However, smaller clinics were introduced, which increased discussion time with medical professionals but radically reduced the number of families attending on any 1 day, resulting in reduced contact with others in the Barth community. As a consequence, Barth Syndrome UK (the UK-based Barth Syndrome charity) has instigated an annual family weekend (where possible), one of which was held between the interviews at the two study timepoints. Therefore, during the time-period of the study, there was a high level of social and instrumental support and social integration, so the present study should be considered within this specific condition and treatment context. This was corroborated by a representative of Barth Syndrome UK (MD) who felt that the findings reported in this manuscript resonated with their experience of supporting families affected by BS.

## Conclusion

The present findings suggest that self-regulation and coping with BS was interpersonal and contingent on parental involvement. Furthermore, parents were aware that their child had a limited energy resource that had to be managed due to the implications of fatigue for daily living. The findings also support previous quantitative work demonstrating that children and parents tend to share a coherent view of BS [7]. However, there is a need for greater awareness from others within the context of social and employment networks in the absence of family support which may have adverse implications for future life choices. With regard to family life these finding serve to validate parents who are sometimes made to feel neurotic and guide the transition process from boyhood to adulthood (for both parents and affected individuals). Furthermore, such knowledge can be used to educate teaching staff around various issues: accepting parents’ involvement where appropriate, and helping young people to self-regulate and advocate for themselves. In addition, employers may use this knowledge as a resource to better understand the unpredictability and severity of fatigue and muscle weakness to accommodate affected individuals by providing flexible working/careers that are manageable, purposeful and enable them to transcend the limitations of a BS diagnosis.

## Data Availability

Data for this study was obtained from participants taking part in an NIHR funded clinical trial and the data cannot be shared on a publicly accessible data repository due to legal and ethical reasons. However, data will be made available for secondary research, conditional on assurance from the secondary researcher that the proposed use of the data is compliant with the with the UK Policy Framework for Health and Social Care Research and MRC Policy on Data Preservation and Sharing regarding scientific quality, ethical requirements and value for money. Please contact cardioman-trial@bristol.ac.uk to discuss any data requests. Data will be made available after the study has been closed and the primary publication is out. It will be made available indefinitely. Only data from patients who have consented for their data to be shared with other researchers will be provided.

## Appendix 1

The participant may enter study if ALL of the following apply: 1. Male aged ≥ 6 years old 2. Clinical diagnosis of Barth syndrome with characteristic abnormality of the L4- cardiolipin/monolysocardiolipin ratio plus identified mutation in the tafazzin gene 3. Under the care of the NHS Barth Syndrome Service (BSS) 4. Stable cardiac condition 5. Able to swallow bezafibrate tablets (similar size to ibuprofen tablets) 4.4.2

### Exclusion criteria

Participant may not enter study if ANY of the following apply: 1. Known hypersensitivity to bezafibrate, to any component of the product or to other fibrates 2. Known photoallergic or phototoxic reactions to fibrates. 3. Hepatic dysfunction and/or liver function tests greater than 2 × normal 4. A shortening fraction of < 90 mL/min) 7. Pre-existing known gallbladder disease. 8. Recent unspecified significant deterioration in general health 9. Prisoners and adults lacking capacity to provide informed consent.
